# Breast Cancer Segmentation Methods: Current Status and Future Potentials

**DOI:** 10.1155/2021/9962109

**Published:** 2021-07-20

**Authors:** Epimack Michael, He Ma, Hong Li, Frank Kulwa, Jing Li

**Affiliations:** ^1^College of Medicine and Biological Information Engineering, Northeastern University, Shenyang 110169, China; ^2^Radiology Department, Affiliated Hospital of Guizhou, Medical Hospital, China

## Abstract

Early breast cancer detection is one of the most important issues that need to be addressed worldwide as it can help increase the survival rate of patients. Mammograms have been used to detect breast cancer in the early stages; if detected in the early stages, it can drastically reduce treatment costs. The detection of tumours in the breast depends on segmentation techniques. Segmentation plays a significant role in image analysis and includes detection, feature extraction, classification, and treatment. Segmentation helps physicians quantify the volume of tissue in the breast for treatment planning. In this work, we have grouped segmentation methods into three groups: classical segmentation that includes region-, threshold-, and edge-based segmentation; machine learning segmentation; and supervised and unsupervised and deep learning segmentation. The findings of our study revealed that region-based segmentation is frequently used for classical methods, and the most frequently used techniques are region growing. Further, a median filter is a robust tool for removing noise. Moreover, the MIAS database is frequently used in classical segmentation methods. Meanwhile, in machine learning segmentation, unsupervised machine learning methods are more frequently used, and U-Net is frequently used for mammogram image segmentation because it does not require many annotated images compared with other deep learning models. Furthermore, reviewed papers revealed that it is possible to train a deep learning model without performing any preprocessing or postprocessing and also showed that the U-Net model is frequently used for mammogram segmentation. The U-Net model is frequently used because it does not require many annotated images and also because of the presence of high-performance GPU computing, which makes it easy to train networks with more layers. Additionally, we identified mammograms and utilised widely used databases, wherein 3 and 28 are public and private databases, respectively.

## 1. Introduction

Breast cancer represents one of the foremost factors behind the death of women worldwide. Hence, early diagnosis and detection increase the probability of recovery and reduce the mortality rate. The World Health Organization (WHO) reported that breast cancer is the most frequently diagnosed disease worldwide, and about 626,700 women die each year due to cancer-related diseases, and more than 2 million new cases were diagnosed in 2018 [1, 2].

However, if it is detected in the early stages, it can drastically reduce the mortality rate and reduce treatment costs, which will be more comfortable for the patients as there will be no need to undergo biopsy. Furthermore, studies have shown that radiologists can misdiagnose breast cancer because of the large number of ultrasound images generated every day, and the number of radiologists available to analyse these medical images is limited. The large number of ultrasound images generated is due to the increase in the number of instances of breast cancer diagnosis; thus, radiologists may become overwhelmed [3].

Different modalities have been used for screening, detection, and diagnosis of breast cancer, such as mammography, magnetic resonance imaging (MRI), positron emission tomography (PET), computed tomography, breast ultrasound [4–6] and [7], and digital breast tomosynthesis, which has been explained in [8, 9].

However, mammography is frequently used because it is considered very efficient for tumour detection in the early stages [10–15], and [16].

The detection and diagnosis of breast cancer in the early stages increases the chances of treatment and reduces mortality by 25.0% [17]. However, tumour detection is based on accurate segmentation of the breast region of interest (ROI). Accurate segmentation of the region of interest is an important part of computer-assisted diagnosis (CAD) [18].

In this study, we reviewed a number of previous research papers related to mammogram image segmentation to detect, extract, or classify breast cancers. An example is [19], which concluded that *k*-means segmentation is frequently used in mammogram segmentation. However, quantitative data have not been presented. Furthermore, *k*-means is based on unsupervised machine learning [20, 21]. In addition, we reviewed the following papers [22–26] and [27].

In the reviewed papers, there were no conclusions drawn regarding the most frequently used techniques for classical segmentation methods and machine learning segmentation. Moreover, for the classical segmentation method, no paper has pinpointed frequently used filters. The quality of segmentation is based on the filter used to remove artifacts from the mammogram images. Furthermore, it has been a big challenge for researchers to find mammogram images, as from the reviewed papers that no conclusion was made as to which database is frequently used for mammogram image segmentation. Owing to these drawbacks, we attempt to address these issues, as highlighted in our contributions below.

- In this survey, we provide a comprehensive study of state-of-art methods for mammogram image segmentation from 1999-2021
- First, we introduce segmentation pipeline used in mammogram images
- Second, we discuss the most frequently used filters to remove noise from mammogram images
- Third, we discuss the publicly and privately available databases for mammogram images and its segmentation metrics and classification
- Finally, we investigate the most frequently used techniques for classical segmentation, machine learning segmentation, and deep learning segmentation.

### 1.1. Role of Segmentation in Mammogram Images

Segmentation in image processing is the process of partitioning an image into multiple regions with the aim of extracting the ROI from an image by identifying the masses in mammograms [28, 29] and [30]. In addition, it is easy to detect abnormalities [31, 32]. However, the detection can be affected by pectoral muscles; hence, artifacts and pectoral muscles should be removed before segmentation. These artifacts and pectoral muscles are not a part of the breast; hence, their presence can misguide the classification algorithms [33, 34] and [35].

Moreover, if segmentation is performed directly from raw images which contain noise and has poor contrast, there is a possibility of oversegmentation and faulty detection of breast tumours. Hence, noise and other local irregularities from noisy images and must be removed using filtering techniques to improve their quality [36, 37]. The aim of segmentation is to extract ROIs with possible masses, which involve partitioning of the mammogram into several nonoverlapping regions [38]. The segmentation methods used for classification of demographic masses can be manual (i.e., traditional), which is based on radiologist experts or fully automated that is based on CDA [39, 40].

However, mammogram segmentation methods can be affected by several factors which can hinder detection of abnormalities in the mammogram images as explained in [41, 42]; these factors include the following:

- Pixel resolution: the ribbons or margins of masses at pixel resolution finer than 200 *μ*m or coarser than 800 *μ*m per pixel are unsuitable for mammography mass classification based on 14 texture features. However, the author concluded that using ribbons of masses with pixel sizes of 400 *μ*m and 800 *μ*m is recommended for a Bayesian classifier based on mammogram mass classification
- Integration scale: higher pixel resolution of mammogram images increases computational times, and when pixel resolution is far too low, it can affect the performance of the texture analysis methods. Hence, the recommended mammogram pixel resolution should be optimal
- Preprocessing methods and feature normalisation: preprocessing mammogram images may affect the performance of texture analysis methods because it effectively changes the grey levels of the mammogram images and normalisation may affect the classification accuracy. The texture feature needs to be normalised to avoid higher numeric ranges from dominating those with lower numeric ranges.

The roles of mammogram images segmentation are as follows:

- Detection: the segmentation helps radiologists detect the breast cancer easily, as the shapes of benign and malignant tumours differ [43, 44]; one tends to be regular, and the other tends to be irregular
- Feature extraction: segmentation is an important step before feature extraction. The principal objective of preprocessing is to process an image such that the results are more suitable than the original image for a specific application [44]. Once the segmentation has been performed, the ROI is used to extract features, which can be extracted using GLCM features from the image by constructing the grey-level cooccurrence matrix of the image [45, 46]
- Classification (accuracy of breast mass): contour segmentation plays an important role in CADx systems for mass classification [47], and an image segmented can be classified as normal, benign, and malignant [3]
- Treatment: the dose for breast cancer treatment depends on the size of tumour, which is an output of mammogram segmentation. Thus, every patient will have a different dose size and different treatment mechanisms. The segmentation of breast and node volumes in the setting of breast cancer treatment includes a definition of irradiation volumes [48, 49].

There are different segmentation methods which are currently used in mammogram segmentation, such as classical segmentation, machine learning segmentation, and deep learning segmentation, as highlighted below. The classical segmentation method has been explained in [46, 50], which depends on digital image processing and mathematics to segment the image which includes the following:

- Edge-based segmentation methods (EBS), such as canny edge detection, active contour, Sobel, energy minimisation, and contour
- Threshold-based segmentation methods (TBS) includes Otsu thresholding, morphological thresholding, adaptive thresholding, manual thresholding, Kittler's optimal thresholding, and global and local thresholding
- Region-based segmentation (RBS), such as watershed, rough set theory, partial region growing, and marker controller.

Machine learning segmentation methods has been proposed in [51–54] and [55], which include the following:

- Unsupervised machine learning methods (USML), such as fuzzy *C*-clustering, *k*-means clustering, novel clustering, and hierarchical *k*-clustering
- Supervised machine learning methods (SML), such as support vector machine (SVM) and extreme learning machine.

Deep learning segmentation methods have been proposed in [56] which include the following:

- Deep learning segmentation (DL), such as SegNet, U-Net, and fully convolutional neural networks (FCN).

Manual segmentation (traditional) is widely used by medical experts to identify tumours in the breast ROI. However, manual work for diagnosis is tedious and requires the skill of medical experts [57], and the number of breast cancer cases increases yearly. Hence, medical experts are overwhelmed and may cause misclassification of breast tumours. Medical experts manually select the abnormal region by comparing to the remaining parts. Moreover, the drawback of this method is that it requires medical experts to accurately select abnormal regions which is time consuming [58], such that it can be performed in real time and is flexible to changes. In addition, assessing the performance of segmentation accuracy is based on the evaluation of medical experts [59], even for automatic segmentation.

### 1.2. Automatic Mammogram Segmentation

Automatic image analysis and automatic segmentation indicate that there is little human intervention (semiautomatic) or without human intervention (fully automatic). Global thresholding, which has traditionally been used over the years to segment images, has been reported to cause misclassification of breast tumours [60]. Recent studies have shown that radiologists' interpretation of mammograms has produced a large number of false-negative cases [61]. To reduce this error, mammograms should be double checked, which increases sensitivity by 9.0% [20]. However, this process is time consuming and costly. Computer-aided diagnosis (CAD) is widely used to assist radiologists in detecting and identifying breast masses [62, 63] and [64]. The adoption of computer-aided diagnosis reduces the number of misclassifications and increases accuracy and time management [65]. Computer-aided diagnosis is considered a second reading for mammograms and has been reported to be more efficient than traditional methods [16]. The generic computer-aided diagnosis system which includes segmentation, feature extraction, and classification stages [66] has been developed to assist medical experts in breast cancer classification. Hence, automatic image segmentation is a very important step in developing computer-aided diagnosis systems [67].

### 1.3. Structure of This Review

**Figure 1:**
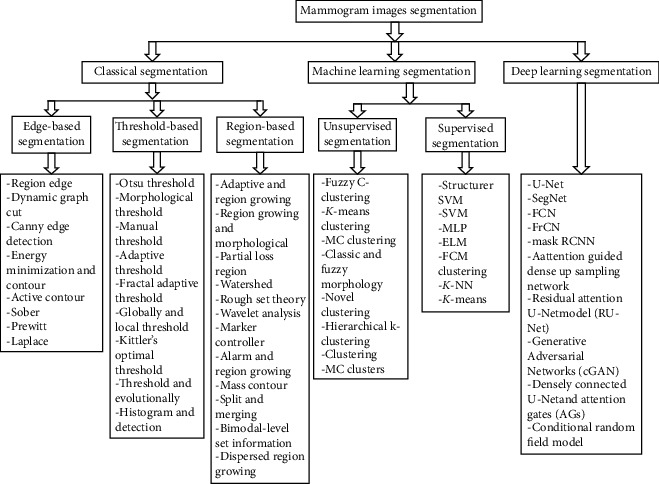
Mammogram image segmentation pipeline.

In this review paper, we have reviewed previous works from 1999 to 2021 which are related to mammogram segmentation based on masses and microcalcifications found in mammogram images. The roles of mammogram image segmentation in image analysis include detention, feature extraction, and classification. Breast cancer is divided into three stages: normal, benign, and malignant. Furthermore, breast cancer can be suspected or detected as either masses, microcalcifications, or architectural distortions that are found in mammogram images. It is worth noting that the scope of this manuscript is to highlight segmentation techniques for breast cancer detection using mammogram images based on masses and microcalcifications. In addition, research findings, mammogram database, current status of mammograms, and future potential are also presented. The mammogram segmentation pipeline is shown in Figure 1. The rest of this paper is structured as follows. “Mammogram Breast Cancer Segmentation Based on Classical Methods” presents a classical segmentation method. “Mammogram Breast Cancer Segmentation-Based Machine Learning Methods” presents the machine learning segmentation method. “Mammogram Breast Cancer Segmentation-Based Deep Learning Methods” presents a deep learning segmentation method. “Methodology Analysis” presents the methodology of the study. Finally, this study is summarised in “Conclusion.”

### 1.4. Merits and Demerits of Mammograms Segmentation Methods

Table 1 summarises the merits and demerits of mammogram segmentation technique presented in Figure 1. These limitations will give insight to the reader to help select the appropriate segmentation technique.

## 2. Mammogram Breast Cancer Segmentation Based on Classical Methods

Some researchers have developed methods for the early detection of breast cancer lesions. The developed methods have been used to segment masses from breast cancer images. The method includes the threshold, active contour model, region-growing, watershed, template-matching, level set, and marker-controlled watershed methods [68, 69] and [70]. The classical segmentation method is based on the pixel values of an image, and it is divided into three groups: region-based segmentation, edge-based segmentation, and threshold-based segmentation [71].

Classical segmentation partitions an image into nonoverlapping regions which have certain attributes [72]. In addition, classical segmentation methods are a first-generation method which is based on low-level techniques, and it requires little information [73]. An overview of classical image segmentation, which is used in mammogram image segmentation, is given below, and a summary is presented in Table 2.

### 2.1. Mammograms Breast Cancer Segmentation-Based Region Method (RM)

Dehghani and Dezfooli [74] presented a method for improving mammogram image preprocessing. The method has two phases: (a) excess image parts were removed using pixel brightness, and (b) the mammogram images were placed in one direction. A total of 60 images acquired from the MIAS database were used to test the algorithm. In addition, the noise from the mammogram images was removed using the threshold limit. The proposed method produced a 99.0% segmentation accuracy.

Senthilkumar et al. [75] formulated a methodology for a region-growing segmentation algorithm to detect breast cancer. A total of 40 mammogram images were acquired from the MIAS database, and a median filter was used to remove noise from the mammogram images. To increase the segmentation accuracy, mammogram images were enhanced using contrast limited adaptive histogram equalisation (CLAHE) and Harris corner. The proposed method produced a segmentation accuracy of 93.0%.

Berber et al. [76] proposed a breast mass contour segmentation method for digital mammograms. The proposed method is based on the classical seed region growth. Furthermore, the method was evaluated using 260 mammogram masses acquired from the Dokuz Eylul Mammography Set (DEMS). The proposed method achieved an accuracy of 95.06%.

Petrick et al. [77] proposed a method which combines adaptive enhancement and region-growing segmentation of breast masses on mammograms. The images were enhanced using density-weighted contrast enhancement, and the noise was removed using a Gaussian filter. The method was tested using 253 mammograms acquired from the University of Michigan Hospital (UMH). The proposed method produced a 98.0% segmentation accuracy.

The automatic detection of breast masses using an optimised region-growing technique was proposed in [78]. Texture features, including grey-level cooccurrence matrix and grey-level run length matrix, were extracted from the segmented images and used as input into the feed forward neural network. The performance of the proposed method was evaluated using 300 mammogram images acquired from the DDSM database, and a Gaussian filter was used to remove the noise from the images. The sensitivity and specificity of the proposed method were 98.1% and 97.8%, respectively. In addition, the segmentation performance was 90.0% based on the Jaccard index.

The automatic breast boundary segmentation of the mammogram method was proposed in [79]. The method used to estimate the skin line and breast segmentation was a modified fast matching algorithm and morphological operators. The proposed method was tested using 136 mammogram images acquired from the mini-MIAS database, and the noise was removed using an alternating sequential filter. The proposed method achieved a sensitivity of 99.2% for the ground truth and 99.0% segmentation accuracy.

Malek et al. [80] proposed a method for growing the seed regions for segmenting mammogram microcalcification images. The proposed method was developed using an automated initial seed point selection algorithm. The algorithm was tested using 50 mammogram images acquired from the National Cancer Society Malaysia (NCSM), and the noise was removed using mathematical morphology. The method was evaluated based on the receiver operator curve (ROC) and produced a 98.0% accuracy.

The detection of microcalcification in digital mammograms based on improving the segmentation method was proposed in [81]. The method uses an improved multiscale morphological gradient watershed segmentation for the automatic detection of clustered microcalcifications in mammograms. The performance of the proposed method was tested using two databases: 322 mammogram images were acquired from the MIAS database, and 100 mammograms were acquired from the local NMR diagnostic centre. The noise was removed using an adaptive median filter. The true positive rates were observed to be 95.3% and 94.0% for the MIAS and NMR databases, respectively.

Isa and Siong [82] proposed the automatic segmentation and detection of masses in digital mammograms. The proposed method is based on region growing in segment mammogram images into two sets: (a) mass pixels set and its surrounding background pixel set and (b) the image contrast was improved by performing image enhancement. The region growing based on local statistical texture analysis was applied to detect and segment the area of interest in the breast mass. The method was evaluated using 322 mammogram images acquired from the MIAS database. The method produced 94.59% sensitivity and had 3.90% false positives per image.

An automated digital mammogram segmentation method using a dispersed region growing and sliding window algorithm was proposed in [83]. The method uses a fully automated technique to detect suspicious masses in mammogram images. The sliding window method was used to remove the pectoral muscles from the mammogram. In addition, a dispersed region-growing algorithm was used to segment the ROI. The mammogram images were acquired from the MIAS database, and the proposed method achieved a 91.3% segmentation accuracy.

Danilo et al. [84] proposed the segmentation and detection of breast cancer in mammogram images. The method uses a set of computational tools to help in the segmentation and detection of mammogram images which contain masses on wavelet analysis and genetic algorithms. The mammogram images were acquired from the DDSM database, and artifacts from the images were removed using a Wiener filter. The detection and segmentation of masses were performed using multiple thresholding techniques, wavelet transform, and a genetic algorithm. The mean and standard deviation were observed to be 79.2 ± 8%.

Podgornova and Sadykov [85] conducted a comparative study of segmentation algorithms to detect microcalcifications on mammogram images. The method was tested using 250 mammogram images acquired from the MIAS database. Watershed, mean shift, and *k*-means segmentation were used in this study. The results show that watershed segmentation was able to detect 18.0% correctly and had 94.0% false detection. In addition, the mean shift method was able to detect 39.22% correctly and had 60.8% false detections. In contrast, *k*-means segmentation detected 42.8% correctly and had 57.2% false detections.

Segmentation of the breast region in digital mammograms and the detection of masses were proposed in [86]. The method uses an automated technique for mammogram image segmentation based on morphology, and the method was capable of removing digitisation noise from mammogram images. The mammogram images were acquired from the mini-MIAS database, and a median filter was used to remove noise from mammogram images. The region detection accuracy was 95.0. The region-growing segmentation and detection of microcalcifications in digitised mammogram images was proposed in [87]. The methods used to detect microcalcifications appear in small clusters within a few pixels in the mammogram images. The pixels, which are high-intensity values, are compared with surrounding pixels and value to determine whether they are benign or malignant. Artificial neural networks (ANNs) were employed to classify mammogram images. Six features were extracted from mammogram images acquired from the MIAS database, and the proposed method achieved an accuracy of 92.5%.

Soukaina et al. [88] proposed breast tumour segmentation and elimination of the pectoral muscle based on hidden Markov and region growing. The scope of the proposed method was to separate the pectoral muscles from mammogram images and feature extraction from breast tumours. The method has two stages: (a) Otsu's thresholding and (b) image classification-based *k*-means. The mammography images were acquired from the MIAS database. The accuracy and error were reported to be 91.92% and 8.07%, respectively.

Mammogram image segmentation using watershed segmentation and classification using *k*-NN classifier was proposed in [89]. The grey-level cooccurrence matrices based on the Halarick texture feature were extracted from 60 mammogram images. The MIAS database was used to test the proposed method, and a Sober filter was used to remove noise. The proposed method produced 83.33% segmentation accuracy.

Wei et al. [90] proposed the segmentation of the breast region in mammograms using watershed segmentation. The method combines two approaches: global thresholding and morphological. The method includes a coarse estimation of the breast ROI and extraction of the position of the breast boundary. A total of 204 mammogram images were randomly chosen from the DDSM mammogram database, and a median filter was used to remove noise. The proposed method produced an accuracy of 95.0%.

The automatic recommendation of the initial mass positions for mass segmentation in mammogram images was proposed in [91]. The method detects the initial position of the mass segment and the segmented mass to radiologists without losing any information. The method developed was based on region growth for breast segmentation. The method was evaluated using mammogram images acquired from the DDSM database. The achieved sensitivity was 78.0% and had 4.0% false positives per image, respectively.

Pei et al. [92] proposed the segmentation of the breast ROI in mammograms using a marker-controlled watershed transform. The method is based on a smoothed morphological gradient image which uses morphological reconstruction and markers. The markers were set using the Otsu method, and 120 mammogram images were acquired from the DDSM database. The results of the proposed method were evaluated by comparing them with the manual borders drawn by medical experts. The mean and standard deviation of the proposed method were reported as 0.93 ± 0.03.

A computer-aided method for segmenting microcalcifications in mammograms using morphological transformations was proposed in [93]. The method is composed of three stages: (a) the detection of microcalcification morphology, (b) noise removal, and (c) mammogram segmentation based on watershed segmentation. A total of 200 mammogram images were acquired from the DDSM database, and the noise was removed using a morphological filter. The proposed method achieved an 80.5% similarity index, 75.7% overlap fraction, 70.8% overlap value, and 19.8% extra fraction.

Soomro and Choi [94] proposed robust active contours for a mammogram image segmentation method. The proposed method detects high-intensity regions in mammograms which are based on a bimodal level set formulation. The method was evaluated using mammogram images acquired from the mini-MIAS database. The performance of the proposed method was measured using precision and recall metrics. The method produced a precision of 96.72%, and the recall was 97.22%.

An automatic method for breast mass segmentation using a marker-controlled watershed algorithm, localised breast masses, and pectoral muscle removal was proposed in [95]. The foreground of the mammogram image and background markers were detected to identify the localised breast tumour ROI. The method was tested on 120 mammogram images acquired from the MIAS database, and the noise was removed using a Sober filter. The proposed method's mass detection rate was reported to be 90.83%, and the receiver operating curve was reported to be 91.3%.

Liu et al. [96] proposed a fully automated scheme for mass detection and segmentation in mammogram images. The method uses a novel technique for automatic mass detection, which is divided into two parts: (a) establishing a search template and (b) adopting template matching to acquire an image. The method employed an adaptive thresholding technique based on the maximum entropy principle to transform the features of the image into ROIs. The region-growing technique was applied to separate mammogram masses from the background. The method was tested on 70 mammogram images acquired from the DDSM database. The proposed method produced a sensitivity of 97.2% and had 1.83 false positives per image.

Hatanaka et al. [97] proposed an automatic method for mass detection in mammograms, which is based on the partial loss of the ROI. The method was tested on 335 Japanese mammogram images, and the noise was removed using a Sober filter. The sensitivity was reported to be 90.0% and had a 0.2 false positive rates per image. In addition, the combination of these two methods improved the true positive to 97.0%.

Shareef [98] proposed a breast cancer detection method based on watershed transformation. The method employed two types of medical images: a total of 33 ultrasound images and 33 X-ray mammogram images. Medical images were acquired from Mosul Hospital Khansa Education (MSKE). The results obtained in this study were found to have 90.47% sensitivity, 75.0% specificity, and 84.848% accuracy for both medical images.

Fauci et al. [99] proposed automatic breast mass segmentation in mammogram images. Segmentation was employed in entire mammogram images, instead of manual partitioning and selection of regions of interest. The pixels, a value which has maximum grey levels, were selected as seeds. The method employed 40 mammogram images acquired from the MIAS database. The proposed method produced a true positive rate of 90.0% and a false positive rate of 1.3.

Jothilakshmi and Raaza [100] proposed an effective method for detecting mass abnormalities and classifying images as benign versus malignant via multi-SVM. The region segmentation method was applied to segmented mammogram images based on the split and merge techniques. Fifty mammogram images were acquired from min-MIAS, and noise was removed using a median filter. The texture features were extracted from the ROI based on a grey-level cooccurrence matrix. A support vector machine was applied to classify the images, and the proposed method achieved 94.0% accuracy.

The region-based contrast enhancement for mammogram images using an improved watershed segmentation was proposed in [101]. The method has three stages: (a) segmentation of the breast ROI and removal of artifacts, (b) identification of the pectoral muscle region using adaptive thresholding, and (c) an improved watershed segmentation were employed to segment the mammogram images. The mammogram images were acquired from the MIAS database, and the classification accuracy achieved was 92.0%.

Qian et al. [102] proposed a wavelet transform and Kalman filtering neural network for region-based segmentation of the mass in mammogram images. The method was used to identify 200 regions of interest in mammograms with similar features. The method uses adaptive modules to improve the computer-aided diagnosis method. These modules used a four-channel wavelet transform with a neural network rather than two-channel decomposition and reconstruction. The receiver operating curve achieved was 93.0% with the adaptive module and 86.0% without the adaptive module.

Mammogram image segmentation based on contour searching and massive lesion classification with a neural network was proposed in [103]. The database consists of 3762 digital mammogram images acquired from the MAGIC-5 collaboration database. The features extracted from mammogram images have two attributes: geometrical information and shape parameters. The features were extracted from the ROI, and these features were used as inputs to the supervised neural network. The ROC was reported to be 85.6Â ± 0.8% for massive lesion detection.

### 2.2. Mammograms Segmentation Based on Threshold Methods (TM)

Omer and Elfadil [104] proposed a segmentation method based on Otsu's threshold. A total of 160 mammograms were acquired from the MIAS database, and noise was removed and enhanced using contrast CLAHE. The multilevel thresholding technique was used to segment the pectoral muscle, and the reported accuracy was 96.0%.

The threshold segmentation method for the detection of masses in mammography was proposed in [105]. This method detects a region of mass using a morphological threshold. The method was tested using 55 mammograms acquired from the mini-MIAS database, and mammograms were enhanced using a median filter and contrast limited adaptive histogram equalisation. The segmentation accuracy was 94.54%, and the false positive rate was 5.45%.

Selvamurugan and Sundararaj [106] proposed a breast cancer detection method using adaptive thresholding. The proposed method exploits coarse and fine segmentations. Coarse segmentation was implemented using histogram fuzzy *c*-segmentation, and fine segmentation was implemented using window adaptive thresholding. The extracted features include area, circularity, correlation of pixel intensity, eccentricity, and entropy of intensity. SVM and *k*-NN were used to classify images as normal versus abnormal. The accuracy achieved was 91.5% for SVM and 70.0% for the *k*-NN classifier.

**Figure 2:**
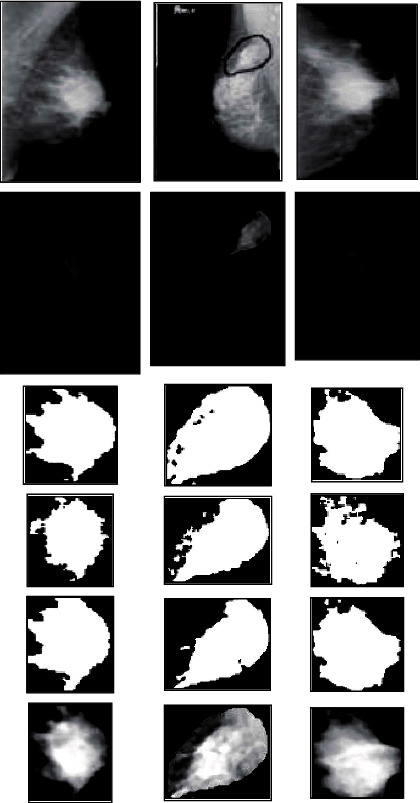
Result of segmented masses. Row (1) shows original images; row (2) shows images after median filtering, cropping, and border removal; row (3) shows the results of the Otsu method; row (4) shows the result of the Otsu method with image smoothing; row (5) shows the result of the Otsu method with Laplacian edge information; and row (6) shows the mass extraction from the original image [107].

The segmentation of masses in digital mammograms using optimal global thresholding using Otsu's method was proposed [107]. The method has three stages: (a) image formation, (b) image preprocessing, and (c) image segmentation. The proposed method was applied to 50 mammography images acquired from the DDSM database, a median filter was used to remove noise, and enhancement was performed using optimal global thresholding, as shown in Figure 2.

**Figure 3:**
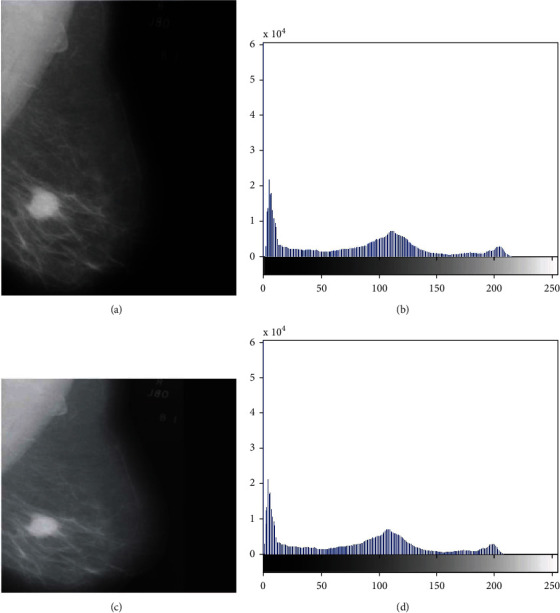
(a) Original image, (b) histogram of the original image, (c) processed image, and (d) histogram of the processed image [109].

**Figure 4:**
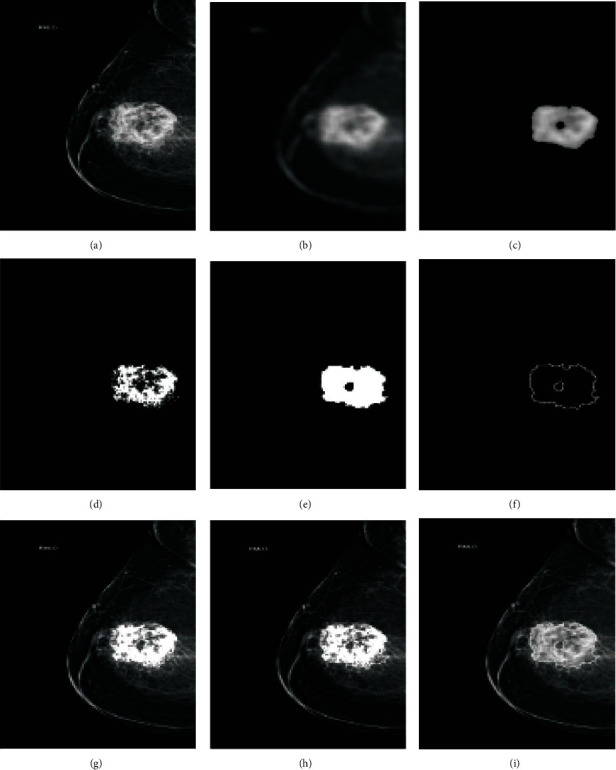
Segmentation and detection result on mammogram image by proposed method: (a) original image, (b) smoothed image, (c) patch image after thresholding, (d) cancer region found in input image in window, (e) region patch found after morphological closing, (f) region boundary using gradient, (g) cancer area detected, (h) cancer area with region segmentation, and (i) proposed segmentation result of cancer in input mammogram image [109].

The preprocessing technique for mammography images was proposed in [108]. The proposed method selects an appropriate enhancement technique to enhance the mammogram. The 322 mammograms were acquired from the MIAS database, and a median filter was used to remove noise. The performance of the algorithm was evaluated using the peak signal-to-noise ratio (PSNR), as shown in Figure 3. Bayati and El-Zaart [109] proposed a novel approach for breast cancer detection and segmentation in mammogram images. The method uses a manual selection threshold parameter and also uses an averaging technique for cancerous tissue detection from two mammogram images. The method was applied using the max-mean and least variance technique for tumour detection, and the results are shown in Figure 4.

**Figure 5:**
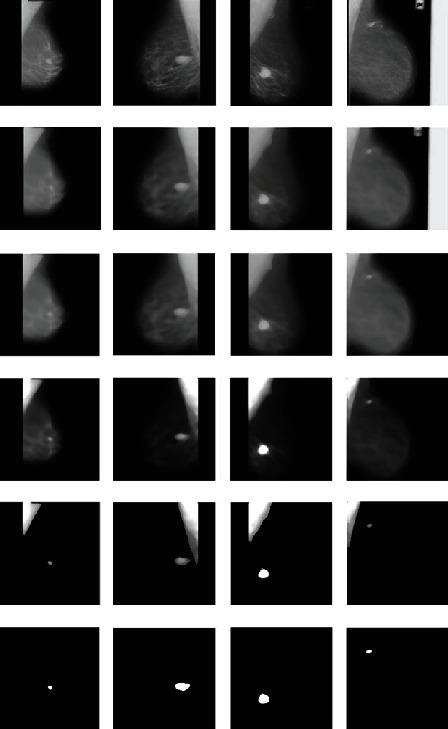
Prepossessing and segmentation results of the proposed method [110].

A novel technique for mammogram mass segmentation using fractal adaptive thresholding was proposed in [110], which was designed to detect breast cancer in early stages. The mammogram images were acquired from the MIAS database, and a median filter was used to remove noise. The images were enhanced to increase their quality. The fractal based on mammogram mass segmentation was able to produce satisfactory results, as shown in Figure 5.

Singh and Veenadhari [111] proposed a breast cancer segmentation method using global thresholding and region merging. Gaussian noise was removed using the Wiener filtering, and image normalisation was performed based on the histogram shrinkage. Global thresholding using Otsu's method was applied to segment the masses from the ROI. The proposed method was implemented and tested in a MATLAB environment on 50 mammogram images to obtain the ROI. The proposed method produced 82.0% accuracy and had an error rate of 18.0%.

The detection of suspicious lesions in mammogram images using adaptive thresholding, which was based on multiresolution analysis, was proposed in [112]. The method utilises a combination of adaptive global and adaptive local thresholding segmentation. The method was tested using 170 mammogram images acquired from the mini-MIAS database, and a morphological filter was used to remove noise. The experimental results achieved were 91.3% sensitivity and 0.71 false positives per image.

Jenefer and Cyrilraj [113] proposed multiclass abnormal breast tissue segmentation using texture features. The texture features were applied at a level set-based bias correction on a mammogram to correct the intensity inhomogeneity. The MIAS database and the proposed method produced 97.0% accuracy and 97.03% specificity.

Neto et al. [114] proposed an automatic segmentation of breast masses in mammogram images using particle swarm optimisation and graph clustering, which is divided into two methods: (a) thresholding and (b) evolutionary algorithms. A total of 100 mammogram images were selected from the DDSM database, and an average filter was applied to remove noise from the mammogram images. The proposed method produced a 95.2% segmentation accuracy.

The automatic segmentation of the pectoral muscles in mammogram images based on global thresholding and weak boundary detection was proposed in [115]. The method identifies and removes pectoral muscles from breast mammograms, followed by convex-hull segmentation. The mammogram images were acquired from the MIAS database, and a median filter was used to remove salt and pepper noise. Global thresholding was used to remove breast tissue from the images. The proposed method achieved 92.86% segmentation accuracy, and 4.97% of the images were segmented to an acceptable level.

Chen and Zwiggelaar [116] proposed a combination of automatic detection of breast boundaries in mammogram images. The developed method is based on histogram thresholding, edge detection, contour growing, polynomial fitting, and the region-growing method. The mammogram images were acquired from two databases, namely, the MIAS database and 248 mammogram images from the EPIC database. Noise was removed using a Gaussian filter. The segmentation results show 98.8% and 91.5% accuracy for MIAS and EPIC, respectively. In addition, the pectoral muscle segmentation accuracies were 92.8% and 87.9% for MIAS and EPIC, respectively.

An accurate segmentation of the breast region of mammogram images was proposed in [117]. The developed method is based on histogram thresholding, morphological filtering, and contour modelling. The method was tested using 20 mammogram images acquired from the DDSM database. The selection of a ROI from mammogram images was based on a manual segmentation technique. The proposed method achieved a 96.0% detection rate, 90.0% acceptable classification, and 55.0% accurate classification.

Liu et al. [118] proposed a muscle segmentation method in mammogram images using the Otsu thresholding and the multiple regression method. The method is based on the position localisation of pectoral muscles in a breast region by combining the Otsu thresholding method and mathematical morphology. The method was tested using the MIAS dataset, and segmented regions were evaluated using the matrix of mean error (ME1), misclassification error (ME2), relative foreground area error (RFAE), extraction error rate (EER), region nonuniformity (NU), and modified Hausdorff distance (MHD) with 1.7188, 0.0083, 0.0056, 0.0134, and 0.8702, respectively.

Sivaramakrishna et al. [119] proposed an automatic segmentation of the mammogram image density. The method is based on Kittler's optimal threshold to estimate breast density on mammogram images. The 32 mammogram images were collected from the breast cancer centre of the Cleveland Clinic Foundation (BCCCF). The Spearman correlation ranged from 0.92 to 0.95, and mammogram density of an average of 6.9% was reported in this study.

Automatic breast segmentation and cancer detection using SVM in mammogram images was proposed in [120]. The method consists of three stages: (a) segmentation of the breast ROI, (b) pectoral muscle removal, and (c) mammogram image classification based on normal versus abnormal. Otsu's segmentation and canny edge detection were applied to remove the pectoral muscles from mammogram images. Mammogram images were acquired from MIAS, and a median filter was used to remove noise. The features were extracted from the ROI based on grey-level cooccurrence matrices. A support vector machine (SVM) classifier was used to classify the mammogram images. The method produced 96.55% accuracy, 96.97% sensitivity, and 96.29% specificity.

The automatic segmentation and classification of masses from digital mammograms were proposed in [121]. The proposed method has three stages: (a) preprocessing to enhance images based on morphological operations and Otsu's thresholding techniques, (b) shape features extracted from the segmented region, and (c) classification performed to classify the segmented shape. The method was tested using 270 mammograms acquired from the Women Health Care Program (WHC) and 142 from the DDSM. The shapes of the segmented masses were classified as round, oval, lobular, or irregular. Round and oval shapes were classified with 100.0% accuracy, while lobular and irregular shapes were 93% accurate, using the ANN based on the WHC database, and had 100.0% accuracy. In addition, for the DDSM database, the method produced accuracies of 100.0% and 91.3%, respectively.

### 2.3. Mammogram Breast Cancer Segmentation Based on the Edge Method (EM)

Cascio et al. [122] proposed mammogram image segmentation using contour searching and breast mass lesion classification using neural networks. The segmentation of mammogram images is based on an edge-based method. A total of 16 features were extracted from segmented images, and 3762 mammogram images were acquired from several hospitals under the MAGIC-5 collaboration. The ROC was found to be 0.862 with 2.8 false positives per image and a sensitivity of 82.0%.

Angayarkanni et al. [123] proposed a dynamic graph cut segmentation of mammogram images. The dynamic graph cut is based on Otsu's segmentation. The developed method improved mammogram images by suppressing unwanted distortions. Mammography images were acquired from the MIAS and DDSM databases. The sensitivity, specificity, positive value, and negative prediction for the proposed method were 98.88%, 98.89%, 93.0%, and 97.5%, respectively.

A fully automatic mammogram breast boundary and mammogram pectoral muscle segmentation was proposed in [124]. The pectoral muscle contour boundary was identified using Canny edge detection, and noise was removed using the median and an anisotropic diffusion filter. Five features were extracted to determine the edge of the breast. A total of 322, 208, and 100 mammogram images were acquired from the MIAS, INbreast, and Breast Cancer Digital Repository (BCDR) databases, respectively. The method achieved Dice similarity coefficients of 98.8% and 97.8% for the MIAS database. It was 98.9% and 89.6% for the INbreast database and 99.2% and 91.9% for the BCDR database.

Mohamed et al. [125] proposed mammogram mass detection and segmentation using cascaded filters. The cascaded filter reduces the resolution of the mammogram images using a Gaussian image pyramid. The method has several steps: (a) developing a breast fat model, (b) removing fat content from mammogram images, and (c) applying a Gabor filter to remove noise and mass detection. The 44 mammogram images were collected from the University Hospital Gasthuisberg Leuven (UHGL). The mammogram ROI was identified using contour processing. The proposed method achieved a sensitivity of 100.0% and had 3.4 false positive per image.

Mello et al. [126] proposed breast segmentation in mammogram images. The developed method establishes the boundaries of the breast ROI. The method uses different image processing methods, namely histogram specification, resampling, histogram adjustment, arithmetic, and morphological. In addition, the method was tested using the mini-MIAS database, and a segmentation accuracy of 97.0% was achieved.

Automatic pectoral muscle segmentation on mediolateral oblique view mammogram images was proposed in [127]. The pectoral edge was estimated using a straight line and was validated based on the location and orientation of the mammogram images. The estimation was performed based on iterative cliff detection of the delineate pectoral margin. In addition, mammogram images of the ROI were generated as a segmentation mask. The method was tested using the MIAS database, and the noise was removed using a median filter. This method produced 83.9% segmentation accuracy.

Ciecholewski [128] proposed the automatic edge detection of breast masses on mammogram images. This method identifies and localises discontinuities in mammogram images. The developed method was tested using 160 mammogram images collected from the mini-MIAS database, and the noise was removed using a 2-D filter. The proposed method achieved 92.5% segmentation accuracy, 93.0% sensitivity, and 85.0% specificity.

Siti et al. [129] proposed a mammogram microcalcification segmentation method based on energy minimisation. Mammogram images were obtained from the National Cancer Society Malaysia (NCSM). The true positive, true negative, false positive, and false negative based on the EDAC segmentation results were evaluated by medical experts. The results show that the ROC was found to be 84.0% based on the enhanced distance active contour, and the area under the curve was 78.0% for the distance active contour.

Mammogram microcalcification segmentation using three edge detection techniques, namely, Sobel, Prewitt, and Laplacian of Gaussian (LoG) edge detection, are proposed in [130]. The method was implemented using enhanced distance active contour model segmentation. The mammogram images were acquired from the NCSM, and the noise was removed using the Adobe Photoshop CS3 software. The ROC shows that the Prewitt edge detection, sober, and LoG were 79.0%, 72.0%, and 71.0%, respectively.

Khalid et al. [131] proposed mass segmentation in mammograms based on energy minimisation and an active contour model. The method uses two approaches: (a) level set theory and (b) minimisation of the active contours energy. The method was tested on the MIAS database, and the criterion was based on the overlapped area ratio between the autosegmented region and manually. The precision of segmentation of masses was 90.0%, and the mean precision was 92.27%.

Mammogram mass classification based on an active contour was proposed in [132]. The method explored three modules: (a) digitisation of mammogram images, (b) mammogram mass segmentation module based on active contour, and (c) a mammogram density classification module. The breast border was determined, an active contour algorithm was employed for mass boundary segmentation, and the sensitivity of the proposed method was 88.0%.

Maitra et al. [133] proposed a breast contour detection method for mammogram images. The method uses a homogeneity enhancement algorithm and an edge detection method. The mammogram images were acquired from the mini-MIAS database, and the noise was removed using a convolution filter. Ground-truth mammogram images and quantitative metrics were evaluated, and the results showed 99.6% completeness, 98.7% correctness, and 98.3% quality.

Al-Najdawi et al. [134] proposed mammogram image enhancement, mass segmentation, and classification. The authors investigated mammogram image enhancement to enhance the performance of the breast ROI, such as the cLare and median filter. The mammogram images were classified as benign, probable benign, possible malignant, probably malignant, and possible benign or malignant. The 1300 mammogram images were collected from the King Hussein Cancer Centre and Jordan Hospital (KHCCJH). The achieved results were 96.2% sensitivity and 94.4% specificity. The classification accuracies for mammogram mass calcification were 94.1% and 81.4%, respectively, and the segmentation accuracy was 90.7%.

### 2.4. Summary of Classical Segmentation Methods

Table 2 shows the summary of classical segmentation works reviewed. The findings of our study revealed that the most frequent classical segmentation method is region growing. Region growing is the most used because region growing has many techniques. In addition, median filter is the most frequently used filter. However, studies show that Gaussian filter has achieved higher accuracy when compared with other filters.

## 3. Mammogram Breast Cancer Segmentation-Based Machine Learning Methods

Machine learning has been used for breast cancer classification. It has several multidisciplinary fields that construct algorithms which can learn and predict from the given data based on features. In this study, we categorised machine learning-based segmentation techniques based on supervised machine learning (SML) and unsupervised machine learning (USML). Some examples of the SML method include support vector machines, and naïve Bayes models. In addition, unsupervised machine learning builds mathematical models from a set of images which contain only inputs, and no other desired output labels are required. Some examples of the USML method include *k*-means and fuzzy *c*-means. The structure of machine learning methods based on mammogram image segmentation is presented in subsections 3.1, and a detailed summary is given in Table 3.

### 3.1. Mammogram Breast Cancer Segmentation Based on Unsupervised Machine Learning (USML)

Automatic breast tumour segmentation using hierarchical *k*-means on mammograms was proposed in [135]. The method uses automatic detection of breast tumours based on valley tracing which helps obtain the optimal number of clusters in mammogram images. The mammogram images were collected from DDSM, and hierarchical *k*-means were used to segment the ROI. The experimental results show that error detection was 61.1% and accuracy was reported to be 38.8%.

Novel mass segmentation in mammogram images was proposed in [136]. The method is based on a mathematical model to detect the location of breast masses. The pixel values were classified using fuzzy *c*-means clustering. The pixel values were divided into three classes: background, initial mass, and boundary. The method was tested using 100 mammogram images acquired from the MIAS database, and a median filter was used to remove the noise. The experimental results show that the mass detection was 98.82%.

Senthilkumar and Umamaheswari [137] proposed a combination of a novel enhancement technique and fuzzy *c*-means clustering technique for breast cancer detection. The method involves computer-aided diagnosis by modifying the local range modification (LRM) as modified (MLRM) for noise removal and enhancement. Mammogram images were acquired using the MIAS database. The combination of MLRM and FCMC yielded an accuracy of 98.1%.

The segmentation of suspicious clustered microcalcifications in mammogram images was proposed in [138]. The method uses a multistage computer-aided diagnosis scheme for the automated segmentation of suspicious breasts. The method consists of three main stages: (a) ROI segmentation, (b) mammogram microcalcification segmentation using a local histogram, and (c) feature extraction. The 98 mammogram images were acquired from the University of South Florida and the Moffitt Cancer Center and Research Institute. The results show that the true positive rate was reported to be 93.2% and had 0.73 false positive clusters per image.

Alam et al. [139] proposed the automatic segmentation of mammogram microcalcification clusters. The segmentation method adopted a series of morphological operations to segment mammogram images, including image decomposition and image interpolation. The mammogram images were acquired from two databases, namely, MIAS and DDSM. A contrast-enhancement filter was applied to remove noise from the mammogram images. The ice metric similarity score was reported to be 0.6192, and the classification accuracies for DDSM and MIAS were 94.48% and 100.0%, respectively.

Hizukuri et al. [140] proposed a computerised segmentation method for mammogram calcifications and maintained their shapes in the CADx schemes. The method was evaluated using 96 mammogram images acquired from the Breastopia Namba Hospital, Miyazaki, Japan (BNHMJ). Eight (8) features were extracted based on the grey-level thresholding technique, and classification was performed using an artificial neural network (ANN). The detection rate and false positives per image were found to be 96.5% and 1.69, respectively. Moreover, the shape-segmentation accuracy was 91.4%.

Salih and Kamil [141] proposed a mammogram image segmentation method based on a fuzzy set and thresholding technique. The method employed a classic morphology and fuzzy morphology. The mammograms were collected from the mini-MIAS database, and a Gaussian filter was used to remove noise. The method produced a 86.0% Dice coefficient, 66.0% recall, and 20.0% precision.

**Figure 6:**
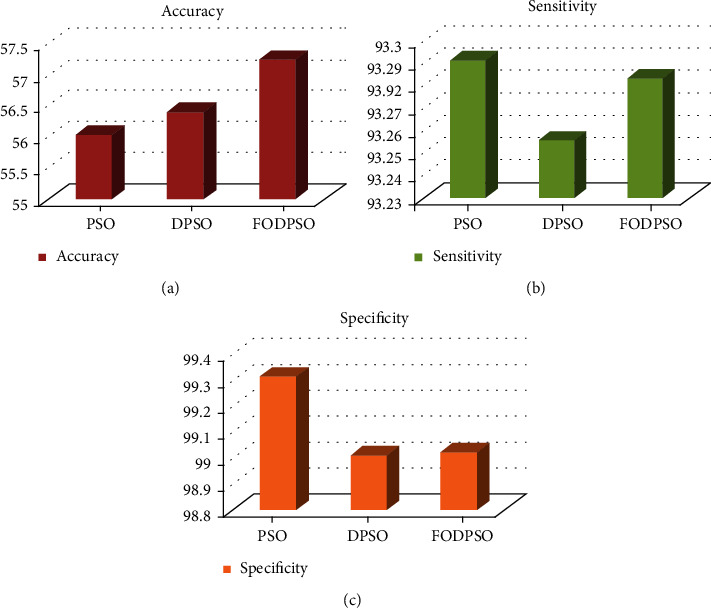
(a) Accuracy, (b) sensitivity, and (c) specificity [142].

Raju and Rao [142] proposed a particle swarm optimisation (PSO) method for mammogram image segmentation based on clustering. The mammogram images were taken from the MIAS, and their performance was evaluated using coefficients, including similarity, accuracy, sensitivity, and specificity. The experimental results show that FCM based on fractional-order Darwinian PSO outperforms other techniques, as shown in Figure 6.

Mughal et al. [143] proposed a deviation analysis for the texture segmentation of breast lesions in mammogram images. The method is based on the colour space and intensity variation. Pixel features were extracted using a colour size histogram from mammogram images. The method was tested using 513 images selected from the MIAS database, and 400 images were acquired from the DDSM database. The salient region, which was based on morphological reconstruction, was adopted to remove noise from mammogram images. The segmentation accuracy achieved was 98.0% for MIAS and 97.0% for the DDSM database.

Letizia et al. [144] proposed a fuzzy technique for microcalcification clustering in digital mammograms. The proposed method is based on fuzzy *c*-means with features. The mammogram images used were acquired from the mini-mammographic (MIAS) database and Policlinic Hospital of Palermo (PHP). Noise was removed using a Laplacian of Gaussian filter. The proposed method achieved an accuracy of 95.0%, sensitivity of 93.0%, and precision of 62.0% for the Policlinic Hospital of Palermo database. In addition, the method achieved an accuracy of 94.0%, sensitivity of 82.0%, and precision of 65.0% for the MIAS database. However, the FP/image remained the same (4.0%) for both databases.

Kulkarn and Shreedhara [145] proposed the identification of breast cancer in mammogram images based on two methods: soft clustering and an artificial neural network (ANN) classifier. The segmentation was performed using fuzzy clustering, and classification was performed using an ANN. Mammogram images were obtained from the MIAS database. The results were categorised into three stages: stage I had an accuracy of 83.3%, stage II had an accuracy of 75.0%, and stage III had an accuracy of 80.0%.

Singh and Kaur [146] proposed a classification of malignant versus benign mammogram microcalcification clusters. The method was applied to two approaches: (a) enhanced ROI using morphological, followed by feature extraction, namely, shape features and cluster texture, and (b) development of a support vector machine classifier. The mammogram images from the DDSM were divided into different sets. The results showed that malignant regions were correctly classified, with a sensitivity of 96.57% and an accuracy of 94.25%. The area under the curve (AUC) for Set2, Set3, and Set4 were 93.83%, 92.58%, and 93.07%, respectively.

The *k*-means clustering method for pectoral muscles and removal of mammogram images was proposed in [147]. The method was tested using 161 mammogram images acquired from the mini-MIAS database, and noise was removed using a 5 × 5 median filter. In addition, mammogram image artifacts were removed using morphological and region-seeded growth. The method was able to remove the pectoral muscle with a 94.4% true positive value and was categorised into three groups: good 90.37%, acceptable 8.07%, and unexpected 1.5%.

Hamissi and Merouani [148] proposed a fully automatic method for the detection of abnormal mammogram masses based on anatomical segmentation of the breast region and classification. The method consists of three stages: (a) removing noise using a 2-D median filter, (b) identifying the breast ROI, and (c) adaptive segmentation based on mean clustering and merging regions of interest. GLCM was used to extract the statistical and textural features. A decision tree was used to classify normal and abnormal cancers from mammogram images acquired from the MIAS database. The proposed method produced a 90.0% sensitivity and 78.0% specificity.

Saleck et al. [149] proposed tumour detection in mammography images using fuzzy *c*-means and GLCM texture features. The proposed method uses an automatic system for mass segmentation in mammograms, using the FCM algorithm. The 18 mammogram images were acquired from the MIAS database, and a median filter was used to remove noise. The proposed method applied a fuzzy *c*-means algorithm to extract the tumour from the ROI, and the FCM input was verified based on the GLCM texture features. The method produced 86.2% sensitivity, 96.4% specificity, and 94.6% accuracy.

### 3.2. Mammogram Breast Cancer Segmentation Based on Supervised Machine Learning (SML)

A novel segmentation of breast masses from mammogram images using a structured support vector machine was proposed in [150]. Mammography images were acquired from the DDSM-BCRP and INbreast databases. The proposed method outperformed other state-of-the-art methods with a computational efficiency of 0.8 s and a Dice index of 87.0%.

Boulehmi et al. [151] proposed a breast mass diagnosis method using a supervised method. The method has four stages: (a) mammogram contrast and enhancement using interpolation, (b) mass segmentation using GGD computing, and (c) Bayesian back-propagation neural network. Features were extracted from mammogram images to detect masses using a neurofuzzy classifier. The MIAS database was used for mass detection, and eight features were extracted based on the mass morphology and texture. The neurofuzzy system was used to classify segmented images as benign versus malignant. The proposed method achieved a 97.08% mass detection rate for GGD analysis, and the Bayesian back-propagation neural network was reported to be 97.0%.

Deep learning, structured prediction based on conditional random field (CRF), and structured support vector machine (SSVM) for mammogram mass segmentation were proposed in [152]. Mammography images were acquired from the DDSM-BCRP and INbreast databases. The Dice index achieved was similar for INbreast and DDSM-BCRP which was 93.0% for CRF and 95.0% for SVM.

A breast density analysis using an automatic density segmentation method was proposed in [153]. The method was validated by comparing it with manual expert annotations with automatic estimations. A total of 130 mammogram images were collected from the Spanish screening program specifications (SSPS) which consists of craniocaudal and mediolateral oblique views, where the noise was removed using a median filter. The study shows that the correlation coefficient of *ρ* = 0.96 between the mammogram density percentage for the left and right breasts, whereas a comparison of both mammogram views showed a correlation of *ρ* = 0.95, based on the SVM classifier.

Cardoso et al. [154] proposed mass segmentation in mammogram images and a cross-sensor compared with deep and tailored features. The authors discuss and compare three models for mass segmentation in mammogram images which include (a) tailored features and boundary computation and (b) second and third models that are based on deep learning features which combine CRF and SSVM. The mammogram images were acquired from two databases, namely, INbreast and DDSM-BCRP, and the cross-sensor performance loss was more than 10.0%.

The segmentation of breast masses in mammogram images was proposed in [155]. The proposed method assesses mammogram density using a multiscale wavelet transform. The density data obtained by processing with wavelet were used to train the multilayer perceptron network (MLP). The trained network was used to detect masses in 19 mammogram images, the true-positive rate (sensitivity) was found to be 68.2%, and the false positive rate was 8.7%.

Cordeiro et al. [156] proposed a segmentation of mammogram images using an extreme learning machine (ELM) for tumour detection for segmentation of tumour breast regions. Mammogram images were obtained from the MIAS database. The ELM classification accuracy was 81.0%.

## 4. Mammogram Breast Cancer Segmentation-Based Deep Learning Methods

Zhu et al. [157] proposed an adversarial deep structured net for mass segmentation from mammograms, which is based on an end-to-end adversarial FCN-CRF network for mammogram mass segmentation. The method was tested using two public datasets: INbreast and DDSM-BCRP. The proposed method achieved a segmentation rate of 97.0%.

Al-antari et al. [158] proposed an integrated computer-aided diagnosis system for the detection, segmentation, and classification of breast masses based on deep learning using You-Only-Look-Once. A regional deep learning approach was proposed to segment the mass based on a full-resolution convolutional network. The method was evaluated using the INbreast database, and the method produced 98.96% mass detection and 97.62% Matthews correlation coefficient (MCC), and the F1 score was 99.24%. In addition, the mass segmentation accuracy based on FrCN was 92.97%, 85.93% for MCC, and 92.69% for Dice and Jaccard similarity was 86.37%. Furthermore, the mass detection and segmentation were classified using CNN, and accuracy achieved 95.64%, 94.78% area under the curve (AUC), 89.91% for MCC, and 96.84% for Dice.

Ravitha et al. [159] developed a deeply supervised U-Net for mass segmentation in digital mammograms (DS-U-Net). The method was tested using the DDSM and INbreast datasets, and the contrast of the images was improved using the cLare filter. The experiments were divided into two groups: whether the images were preprocessed or not. It was found that the experiments when the preprocessing was performed achieved good results compared to when there was no preprocessing. The method achieved 82.70% of Dice, 85.70% of Jaccard coefficient, 99.70% accuracy, 83.10% sensitivity, and 99.80% specificity based on preprocessing.

Tree-reweighted belief propagation using deep learning potentials for mass mammogram segmentation was proposed in [160]. The method was implemented using a conditional random field model (CRF), and the evaluation was tested using the INbreast and DDSM-BCRP databases. The method uses statistical learning methods, and the mass segmentation error is reduced based on tree-reweighted belief propagation. The proposed method achieved an 89.0% Dice index in 0.1.

Shen et al. [161] proposed the mixed-supervision-guided and residual-aided classification U-Net model (ResCU-Net) for joint segmentation and classification of mammogram images. The mammogram images were taken from the INbreast dataset, and convolutional filters were employed to remove noise. The proposed MS-ResCU-Net model achieved an accuracy of 94.16%, sensitivity of 93.11%, specificity of 95.02%, DI of 91.78%, Jac of 85.13%, and MCC of 87.22%, while ResCU-Net correspondingly achieved 92.91%, 91.51%, 94.64%, 90.50%, 83.02%, and 84.99%.

**Figure 7:**
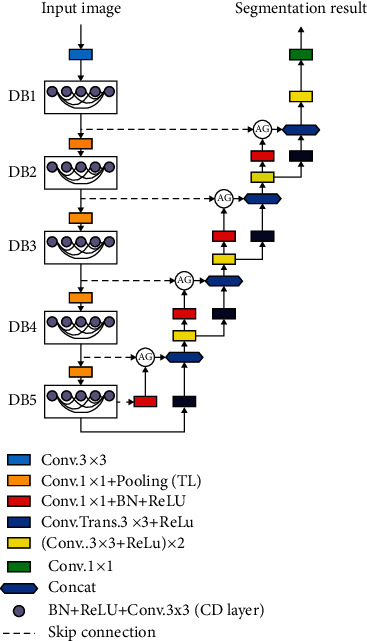
U-Net mode [162].

The attention dense U-Net for automatic breast mass segmentation in mammogram images was proposed in [162]. This method uses a fully automatic method based on deep learning for breast mass segmentation. This method combines densely connected U-Net and attention gates (AGs) for mammogram segmentation. Additionally, this method was tested using the DDSM database, and the experimental results showed that dense U-Net integrated with AGs outperformed other methods. The method achieved 82.24% F1 score, 77.89% sensitivity, and overall accuracy of 78.38%, and the U-Net mode structure is presented in Figure 7.

Min et al. [163] proposed mammographic CAD for simultaneous mass detection and segmentation based on pseudocolour mammograms and mask RCNN. The method was tested using a dataset obtained from the INbreast database, and morphological filters were used to enhance the mammogram images. The DSI achieved for mass segmentation was 0.88Â ± 0.10, and GMs and mask RCNN yielded an average TPR of 0.90Â ± 0.05.

Al-Antari et al. [164] proposed a full-resolution convolutional network (FrCN), which is a novel segmentation model to segment mammogram images. In addition, three conventional deep learning models, namely, regular feedforward CNN, ResNet-50, and InceptionResNet-V2, were adopted to classify the detected and segmented breast lesions as either benign or malignant. Mammography images were acquired from the INbreast database. The results of the breast lesion segmentation based on FrCN achieved an overall accuracy of 92.97%, 85.93% for MCC, 92.69% for Dice, and 86.37% for the Jaccard similarity coefficient.

Abdelhafiz et al. [165] proposed a convolutional neural network for automated mass segmentation in mammography. The model is based on the architecture of the semantic segmentation U-Net model which was originally invented for biomedical image segmentation tasks. The proposed method was tested on four databases, CBIS-DDSM, INbreast, UCHCDM, and BCDR-01, and noise was removed using an adaptive median filter. The proposed U-Net model achieved a mean Dice coefficient index of 95.10% and a mean IOU of 90.90%. Moreover, there is an improvement in the results when using data augmentation, as the Dice coefficient index increases from 92.20% to 95.10% and 85.0% to 90.90%, respectively.

Saffari et al. [166] proposed fully automated breast density segmentation based on conditional generative adversarial networks (cGAN) and classification using deep learning. The cGAN network was applied to segment the dense tissues in mammogram images. The performance was tested using 410 images of 115 patients acquired from the INbreast dataset, and a median filter was used to remove noise. The results achieved based on cGAN segmentation were as follows: accuracy of 98.0%, Dice coefficient of 88.0%, and Jaccard index of 78.0%.

Singh et al. [167] proposed breast tumour segmentation and shape classification in mammograms using the cGAN and convolutional neural networks. The cGAN segments a breast tumour within an ROI in a mammogram. DDSM data containing 2620 mammography images and the INbreast dataset, which contained a total of 115 cases (410 mammograms), were used to test the performance. Morphological operations were used to remove noise from the mammogram images. The proposed cGAN model achieved a Dice coefficient of 94.0% and an intersection over union (IoU) of 87.0%.

Ahmed et al. [168] developed semantic segmentation for breast cancer using two deep neural networks, including mask RCNN and DeepLab. Two datasets, MIAS and DDSM, were employed to evaluate the performance of the proposed method, and noise was removed using a Savitzky Golay filter which is based on edge smoothness. The method achieved an AUC of 98.0% for mask RCNN and 95.0% for DeepLab. However, the mean precision for the segmentation task was 80.0% and 75.0%.

Abdelhafiz et al. [169] proposed a residual deep learning system for mass segmentation-based residual attention U-Net model (RU-Net), and classification was performed based on the ResNet classifier (RU-Net). Three datasets were used to evaluate the performance of the proposed method, DDSM, BCDR-01, and INbreast datasets, and noise was removed using the cLare filter. The proposed model achieved a mean test pixel accuracy of 98.0%, a mean Dice coefficient index (DI) of 98.0%, and mean IOU of 94.0%.

Hossain [170] proposed microcalcification segmentation using a modified U-Net segmentation network from mammogram images. The proposed method was trained with images acquired from the DDSM database, and noise was removed using the Laplacian filter. The method was divided into five stages: image preprocessing, segmentation of breast regions, extraction of suspicious patches, selection of positive patches, and training of the segmentation network. The method produced 98.50% of the *F*-measure and a 97.80% Dice score. Additionally, the Jaccard index was observed to be 97.40%, and the average accuracy of the proposed method was 98.20%.

Sun et al. [171] developed a novel attention-guided dense-upsampling network for breast mass segmentation in whole mammogram (AUNet). AUNet is an asymmetrical encoder-decoder structure which is an effective upsampling block and attention-guided dense-upsampling block (AU block). The method was tested using two publicly available datasets, CBIS-DDSM and INbreast, and produced an average Dice similarity coefficient of 81.80% for CBIS-DDSM and 79.10% for INbreast.

Tsochatzidis et al. [172] proposed a modified convolutional layer of a CNN based on the U-Net model. The method was evaluated using two datasets: DDSM-400 and CBIS-DDSM. The method achieved a diagnostic performance of 89.8% and AUC of 86.20% based on ground-truth segmentation maps and a maximum of 88.0% and 86.0% for U-Net-based segmentation for DDSM-400 and CBIS-DDSM, respectively.

Salama and Aly [173] proposed a deep learning model for mammography image segmentation and classification. The modified U-Net model was used to segment the breast area from the mammogram images. The model was tested using three mammographic datasets: MIAS, DDSM, and CBIS-DDSM. The proposed model achieved 98.87% accuracy, 98.88% area under the curve (AUC), 98.98% sensitivity, 98.79% precision, and 97.99% F1 score on the DDSM datasets.

Li et al. [174] improved breast mass segmentation in mammograms with conditional residual U-Net (CRU-Net) by integrating residual learning and probabilistic graphical modelling with standard U-Net to improve the performance of U-Net. The CRU-Net method was evaluated using two publicly available datasets, INbreast and DDSM-BCRP. The CRU-Net achieved Dice Index a DI of 93.66% for INbreast and a DI of 93.32% for the DDSM-BCRP dataset.

Bhatti et al. [175] developed multidetection and segmentation of breast lesions based on mask RCNN-FPN. The method is based on a regional learning technique known as a masked regional convolutional neural network which is embedded with a feature pyramid network. The method involved training on DDSM and testing on the INbreast database. The model achieved a mean average precision of 84.0% for multidetection and 91.0% segmentation accuracy.

Zeiser et al. [176] proposed the U-Net segmentation of masses on mammogram images using data augmentation. The model was tested using 7989 mammogram images acquired from the DDSM database. The model achieved a sensitivity of 92.32%, specificity of 80.47%, accuracy of 85.95%, Dice coefficient index of 79.39%, and AUC of 86.40%.

### 4.1. Summary of Mammograms Breast Cancer Segmentation Based on Machine Learning Methods

A summary of the machine learning methods is presented in Table 3. The findings of our study revealed that supervised machine learning is more frequently used than unsupervised methods. A detailed analysis is provided in Section 5.3.

## 5. Methodology Analysis

### 5.1. Importance of Mammogram Segmentation as Applied in Medical Field

Segmentation techniques have been used in computer vision in medical images for the detection and identification of abnormalities in images. Segmentation helps doctors or physicians quantify the volume of tissue, locate pathology, diagnose, study anatomical structures, and plan for treatment [177]. The segmented parts will make it easy for medical experts to draw conclusions about whether a particular medical image is normal or abnormal. Detection and identification of diseases can be applied to different medical images. However, the scope of this paper covers only one type of disease, breast cancer, and one type of mammography image modality. A detailed analysis of mammogram image segmentation, which is based on classical, machine learning, and deep learning, is illustrated in subsections 5.2.

### 5.2. Analysis on Classical Methods

Table 2 revealed that region-growing segmentation is frequently used. Furthermore, the study shows that median filter is the most frequently used compared to other filters. Moreover, the region-growing method has outperformed other methods. The highest accuracy achieved was 99.0%, based on threshold limit [74]. The highest threshold segmentation accuracy achieved was 100.0%, and the filter used was the morphological filter [121]. Furthermore, for the edge segmentation method, the highest sensitivity achieved was 100.0%, and the filter used was the Gabor filter [125].

### 5.3. Analysis on Machine Learning-Based Methods

The machine learning described in Table 3 is divided into two parts: supervised machine learning and unsupervised machine learning. In addition, most of the reviewed papers did not mention filters used to remove noise. Moreover, Table 3 reveals that unsupervised machine learning methods are frequently used compared to supervised methods. Additionally, the study showed that the highest detection achieved for supervised machine learning was 99.82% [136].

### 5.4. Analysis on Deep Learning-Based Methods

Table 4 summarised deep leaning segmentation method applied on mammogram, such as SegNet, U-Net, DeepLab, FCN, GAN, CRF, CRU-Net, and FrCN. However, U-Net segmentation was originally designed for medical images and does not require many annotated images, whereas many deep leaning models require many such images. Furthermore, most of the reviewed papers did not mention the filters used, because it is possible to train deep learning model without any preprocessing and postprocessing modules. Moreover, the most frequently used segmentation is U-Net, and it also outperformed other models as it achieved a Dice of 98.87% [173].

### 5.5. Analysis on Mammography Databases

The public mammogram images databases used in this study include the following:

- Mammographic Image Analysis Society (MIAS): the database consists of 322 digitised films, as well as a radiologist's ground truth [178]
- Digital Database for Screening Mammography (DDSM): the database consists of 2500 cases and contains suspicious areas [179]
- INbreast database: the database consists of 118 cases (410 images) [154, 158, 159] and [180].

The private mammogram databases available include the following:

- Dokuz eylul mammogram set (DEMS): the database consists of 260 mammogram images [76]
- University of Michigan Hospital (UMH): the database consists of 253 mammogram images [77]
- Local NMR Diagnostic Centre, Hubli (NMR): the database consists of 100 mammogram images [81]
- National Cancer Society Malaysia (NCSM): the database consists of 50 mammogram images [80, 129, 130] and [181]
- Japan database: the database contains 335 Japanese mammogram images [97]
- Mosul Hospital Khansa Education (MSKE): the database consists of 33 ultrasound images and 33 X-ray mammogram images [98]
- European Prospective Investigation of Cancer (EPIC): the database consists of 248 MLO mammogram images [116]
- Breast Centre of the Cleveland Clinic Foundation (BCCCF): the database consists of 32 normal mammogram images [119]
- MAGIC-5 collaboration: the database consists of 3762 digitised mammogram images [103, 122]
- Women Health Care Program (WHC): the database consists of 270 mammogram images [121]
- King Hussein Cancer Centre (KHCC) and Jordan Hospital(JH): the database consists of 1317 mammograms [134]
- Breastopia Namba Hospital, Miyazaki, Japan (BNHMJ): the database consists of 96 mammogram calcification images [140]
- Policlinic Hospital of Palemrmo (PHP): the database consists of 39 mammogram images [144]
- Oncology Moulay Abdellah, Rabat, Morocco: the database consist of 20 mammogram images [158]
- Spanish Screening Programme Specifications (SSPS): the database consists of 130 mammogram images [159]
- University Hospital Gasthuisberg Leuven (UHGL): the database consists of 44 mammogram images [125]
- Hospital Universitari sant Joan Reus, Spain: the database consists of 14,000 mammogram which contains the craniocaudal and mediolateral views [182]
- Community Hospital in San Francisco, California: the database consists of 158,554 mammograms which includes 106,405 screening mammograms and 52,149 diagnostic mammograms images [183]
- National Mammography Database: the database consists of 3,181,437 mammogram images [184]
- Department of Radiology, Stanford University Medical Centre and Radiology Residency Program: the database consists of 8682 mammogram calcification images [185].
- Froedtert and Medical College of Wisconsin: the database consists of 62,219 screening and diagnostic mammogram images [186]
- New Hampshire Mammography Network of the National Cancer Institute-Sponsored Breast Cancer Surveillance Consortium: the database consists of 118,549 mammogram images [187]
- Department of Radiology, Massachusetts General Hospital: the database consists of 3665 which included 1502 images combined tomosynthesis and 2163 mammography images [188]
- Department of Radiology, University of Michigan Medical Centre: the database consists of 253 mammograms which containing biopsy-proved masses [189]
- Grid Platform for Computer-Assisted Library for Mammography (GPCalma): the database consists of 3369 mammographic images [190].
- Mammography Image Reading for Radiologists and Computers Learning Database (MIRaCLe): the database consists of 204 mammographic images [191]
- Mammographic Image Database for Automated Analysis (MIDAS): the database consists of 600 mammographic images [192]
- Department of Radiology at the University of Chicago: the database consists of 200 mammographic images [193].

The findings of our study revealed that the MIAS database is frequently used by researchers for classical segmentation and machine learning segmentation methods.

### 5.6. Current Status on Mammogram Breast Segmentation Methods

Currently, segmentation methods are categorised into three groups: manual segmentation which is based on the annotation of medical experts, semiautomatic segmentation, and fully automatic segmentation. Furthermore, these three methods depend on medical experts to draw conclusions about whether the segmented parts are benign or malignant. In addition, the segmentation methods discussed in this study can be used in other modalities. However, no pectoral muscle removal was observed for other imaging modalities.

### 5.7. Potential Future for Mammogram Breast Cancer Segmentation Methods

As mentioned earlier, the segmentation methods discussed are divided into three groups, namely, classical segmentation, machine learning methods, and deep learning methods, and focus only on one type of medical image. The discussed methods can be used to detect and identify breast cancer in other imaging modalities, such as MRI and ultrasound. Furthermore, the segmentation methods discussed in this paper revealed accurate segmentation, detection, and identification of mammogram images, depending on the annotation from medical experts. However, machine learning methods require images which have been marked by medical experts and have been proven by a pathology report whether it is benign or malignant. The future potential is to determine the accuracy of the segmentation methods without annotation from medical experts. In our future work, we plan to divide the segmentation methods based on mass segmentation and microcalcification segmentation in mammogram images.

### 5.8. Recommendation

The expectation of breast cancer patients is to receive correct and precise results. Radiologists sometimes generate wrong results by predicting that a patient has cancer, while in reality, the patient may not have cancer. This scenario can occur because of the large number of mammogram images which are generated every day, while the number of radiologists to analyse them is limited. This scenario has implications for patients, because if radiologists found that a patient has no cancer, but in reality she or he has cancer, it can lead to unnecessary large costs in the future and sometimes death if cancer is detected in the late stage. On the other hand, if radiologists diagnose a patient with breast cancer, but in reality she or he does not have breast cancer, the patient might incur unnecessary costs as well as painful treatments due to the biopsy and stress. Hence, to address this issue, we recommend that collaboration between radiologists and artificial intelligence experts should be increased to reduce the workload of radiologists.

## 6. Conclusion

Despite significant progress in medical image segmentation, the impact of segmentation still does not meet the needs of practical applications. Mammogram image segmentation is a cross-disciplinary field between physicians and machine learning experts. Furthermore, breast tumours are complex, and artificial intelligence experts do not understand the clinical needs. In addition, physicians do not understand how artificial intelligence works; hence, specific clinical needs are not met. Collaboration between physicians and machine learning experts should be increased to meet these clinical needs. This will assist machine learning experts in developing deep learning models that meet clinical needs, resulting in a reduced workload for physicians. In this paper, an overview image segmentation was grouped into three categories: classical segmentation, machine learning segmentation, and deep learning methods. Furthermore, the classical method is subcategorised into three parts: (a) region-growing segmentation, (b) threshold segmentation, and (c) edge segmentation. In addition, machine learning methods are subcategorised into two parts: unsupervised machine learning and supervised machine learning. The reviewed works are presented based on the year of publication, as shown in Tables 2–4. The findings of our study revealed that region-based segmentation is frequently used for classical methods, and the most frequently used technique is region growing. In addition, the median filter and MIAS database are frequently used in the classical segmentation methods. In contrast, unsupervised machine methods are frequently used for machine learning segmentation, compared to supervised machine learning. U-Net is mostly frequently used based on deep learning models, because the method was developed specifically for medical image data and does not require many annotated images. Furthermore, it is possible to train networks with more layers owing to the presence of high-performance GPU computing. Finally, we identified mammogram databases which are widely used.

## Figures and Tables

**Table 1:** Summary of merits and demerits of mammograms segmentation methods.

Category	Merits	Demerits
Edge-based segmentation methods	Works well when an edge is prominent	Sensitivity to noise
		Reduces overall contrast in mammograms
	Easy to find locally edge orientation	
		Produce unsatisfactory results when it detects fake and weak edges in mammograms
		Not suitable for mammogram images having smooth edges
Threshold-based segmentation methods	Simple and easy to implement	It is not applicable if the tumour area ratio is unknown
		Sensitive to noise in mammograms
	Faster	
		Gives poor results when mammograms have low contrast
	Inexpensive	
		Difficulties to fix the threshold value if the number of regions increases
		Not easy to process the mammogram whose histograms are nearly unimodal
Region-based segmentation	Connected regions are guaranteed	Causes over segmentation if mammograms are noisy
	Multiple criterion and gives good results with less noise	Cannot distinguish the shading of the real mammograms
		Time consuming due to the high resolution of mammograms
		Not suitable for noisy mammograms
		Seed point must be selected

Unsupervised machine learning methods	Few data are required	Number of clusters must be defined
	Easy to implement	
	Prior information required	
	Automatic segment masses	
Supervised machine learning methods	Easy to detect error	Knowledge about the mammogram to be segmented is required
		Require lab data

Deep learning methods	Solve complex tasks	Limited annotated data
	Required unlabeled data	Time consuming during training
		Expensive because it requires higher computational machines
	Produce accurate results	

**Table 2:** Summary of reviewed works related to classical segmentation in mammogram image.

Subcategory	Related works	Year	Technique	Filter	Database	Evaluation metric
RM	[77]	1999	Adaptive and region growing	Gaussian	UMH	98.0% accuracy
RM	[102]	2001	Region growing	Kalman	DDSM	93.0% ROC with adaptive module and 86.0% ROC without the adaptive module
RM	[97]	2001	Partial loss of region	Sober	Japanese	97.0% true positive
RM	[99]	2004	Region growing		MIAS	90.0% TPR, and 1.3 FTR per image
RM	[103]	2004	Contour searching		MAGIC-5	85.6 ± 08% ROC
RM	[87]	2005	Region growing	ANN	MIAS	92.5% accuracy
RM	[90]	2006	Morphological algorithm	Median	MIAS	95.0% detection rate
RM	[75]	2010	Harris corner	Median	MIAS	93.0% segmentation accuracy
RM	[91]	2010	Region growing		DDSM	78.0% sensitivity and 4.0% false positive
RM	[92]	2010	Watershed	Morphological	DDSM	Mean standard 0.93 ± 0.03
RM	[74]	2011	Thresholding	Median	MIAS	99.0% segmentation accuracy
RM	[82]	2012	Region growing	Contrast	MIAS	94.59% sensitivity and 3.90 false positive
RM	[86]	2012	Morphological	Median	MIAS	95.0% detection rate
RM	[96]	2012	Region growing	Adaptive	DDSM	97.2% sensitivity and 1.83% false positive
RM	[80]	2012	Seed point selection	Mathematical morphology	NCSM	98.0% accuracy
RM	[81]	2013	Morphological gradient watershed	Adaptive median	MIAS and NMR	95.3% positive for MIAS and 94.0% for NMR
RM	[101]	2013	Improved watershed	Median	MIAS	92.0% accuracy
RM	[76]	2013	Otsu	Morphological	DEMS	95.06% accuracy
RM	[95]	2014	Marker-controlled watershed	Sober	MIAS	90.83% detection rate and 91.3% ROC
RM	[84]	2014	Wavelet and genetic algorithm	Wiener	MIAS and DDSM	79.2 ± 8% mean and standard deviation
RM	[98]	2014	Watershed transformation		MSKE	90.47% sensitivity, 75.0% specificity, and 84.848% accuracy
RM	[79]	2015	Morphological operators	Alternating sequential filter	MIAS	99.2% sensitivity and 99.0% accuracy
RM	[83]	2017	Region growing	Sliding window	MIAS	91.3% accuracy
RM	[100]	2017	Region growing	Median	MIAS	94.0% accuracy
RM	[93]	2017	Watershed	Morphological	DDSM	80.5% similarity index, 75.7% overlap value
RM	[94]	2017	Bimodal-level set formulation		MIAS	96.72% precision and 97.22% recall
RM	[88]	2018	Hidden Markov and region growing		MIAS	91.92% accuracy and 8.07% error
RM	[89]	2018	Watershed combined with *k*-NN	Sober	MIAS	83.33% accuracy
RM	[78]	2018	Region growing	Gaussian	DDSM	98.1% sensitivity, 97.8% specificity, and 90.0% accuracy
RM	[85]	2019	Watershed		MIAS	94.0% false detection and 18.0% positive detection

TM	[118]	2001	Otsu thresholding	Morphological	MIAS	1.7188 ME1, 0.0083 ME2, and 0.8702 MHD
TM	[120]	2001	Otsu	Median	MIAS	96.55% accuracy, 96.97% sensitivity, and 96.29% specificity
TM	[113]	2011			MIAS	97.0% accuracy, 97.03% specificity, and 97.0% sensitivity
TM	[117]	2012	Histogram thresholding	Morphological	DDSM	96.0% detection rate and 90.0% accuracy
TM	[119]	2012	Kittler's optimal thresholding		BCCCF	92.0% to 95.0% Spearman and 6.9% average density
TM	[109]	2013	Otsu	Median		
TM	[108]	2014	Rough set theory	Median	MIAS	
TM	[107]	2014	Otsu thresholding	Morphological and median	DDSM	
TM	[114]	2014	Threshold and evolutionary	Average	DDSM	95.2% accuracy
TM	[110]	2014	Otsu	Median	MIAS	
TM	[115]	2015	Global threshold	Median	MIAS	92.86% accuracy and acceptable level of 4.97%
TM	[111]	2015	Global thresholding and merging	Wiener		82.0% accuracy and 18.0% error detection
TM	[105]	2016	Morphological threshold	Median	MIAS	94.54% accuracy and 5.45% false identification
TM	[106]	2016	Adaptive threshold			91.5% accuracy for SVM and 70.0% accuracy for *k*-NN
TM	[121]	2016	Otsu	Morphological	WHC and DDSM	100.0% accuracy for WHC and 91.30% for DDSM
TM	[104]	2017	Otsu	Clahe	MIAS	96.0% accuracy
TM	[116]	2017	Histogram and edge detection	Gaussian	MIAS and EPIC	98.8% accuracy (MIAS) and 91.5% (EPIC)
TM	[112]	2018	Adaptive global and local threshold	Meteorological	MIAS	91.3% sensitivity and 0.71% false positive

EM	[128]	2004	Edge	2-D	MIAS	92.5% accuracy, 93.0% sensitivity, and 85.0% specificity
EM	[122]	2006	Edge		MAGIC-5 collaboration	86.20% ROC and 82.0% sensitivity
EM	[126]	2009	Histogram	Morphological	MIAS	97.0% accuracy
EM	[133]	2011	Active contour	Binary homogeneity	MIAS	99.6% CM, 98.7% CR, and 98.3% quality
EM	[131]	2011	Energy minimisation and contour		MIAS	90.0% accuracy and 92.27% precision
EM	[134]	2011	Edge	Median	KHCCJH	94.1% accuracy (CC), 81.4% MLO, and 90.0% accuracy
EM	[130]	2011	Sobel, Prewitt, Laplacian	Adobe Photoshop	NCSM	79.0% AUC for Sobel, 72.0% Prewitt, and 71.0% Laplacian
EM	[127]	2012	Edge	Median	MIAS	83.9% accuracy
EM	[132]	2014	Active contour			88.0% sensitivity
EM	[123]	2015	Dynamic graph cut		MIAS and DDSM	98.88% sensitivity, 98.89% specificity, and 93.0% for negative values
EM	[124]	2015	Canny edge detection	Median	MIAS, INbreast, and BCDR	98.8% Dice boundary of 97.8% MIAS, 98.9% for boundary 89.6% INbreast, and 99.2% for boundary, and 91.9% BCDR
EM	[135]	2017	Cascade	Gabor	UHGL	100.0% sensitivity and 3.4% false positives
EM	[129]	2017	Edge		NCSM	84.0% AUC

**Table 3:** Summary of reviewed works on supervised and unsupervised machine learning.

Subcategory	Related works	Year	Technique	Filter	Database	Evaluation metric
USML	[148]	2012	Clustering	2-D median	MIAS	90.0% sensitivity and 78.0% specificity
USML	[140]	2012	Microcalcification clusters		BNHMJ	91.4% segmentation accuracy, false positive 96.5%
USML	[142]	2013	FCM clustering	Morphological	MIAS	
USML	[138]	2013	Microcalcification clusters		DDSM	93.2% positive rate and 0.73 false positive
USML	[147]	2014	*k*-means	5 × 5 median	MIAS	94.4% sensitivity
USML	[145]	2015	Fuzzy *c*-means		MIAS	83.3% for class 1, 75.0% class 2, and 80.0% class 3 accuracy
USML	[149]	2017	FCM clustering		MIAS	86.2% sensitivity, 96.4% specificity, and 94.6% accuracy
USML	[139]	2018	MC clusters	Morphological	DDSM and MIAS	94.48% classification accuracy for DDSM and 100.0% for MIAS
USML	[136]	2018	Fuzzy *c*-means clustering		MIAS	98.82% detection
USML	[137]	2018	*c*-means clustering		MIAS	98.1% accuracy
USML	[141]	2018	Classic and fuzzy morphology	Gaussian	MIAS	0.86 Dice, 66.0% recall and 20% precision
USML	[144]	2018	*c*-means	LoG	MIAS and PHP	95.0% accuracy PHP and 94.0% MIAS
USML	[143]	2018		Morphological	DDSM and MIAS	98.0% accuracy for MIAS and 97.0% for DDSM accuracy
USML	[135]	2018	Hierarchical *k*-means clustering		DDSM	38.8% accuracy and 61.1% testing error
USML	[146]	2018	MC clusters	Morphological	DDSM	96.57% sensitivity and 94.25% accuracy

SML	[155]	2011	MLP		DDSM	68.2% sensitivity and 8.7% false positive per image
SML	[156]	2012	ELM		MIAS	81.10% of accuracy
SML	[150]	2015	Structure SVM		DDSM and INbreast	87.0% Dice
SML	[152]	2015	SSVM and CRF		DDSM and INbreast	93.0% accuracy using CRF and 95.0% accuracy using SVM
SML	[153]	2015	SVM	Median filter	SSPS	96.0% correlation
SML	[151]	2016	GGD and Bayesian back propagation		MIAS	97.08% detection for GGD and 97.0% for Bayesian
SML	[154]	2017	CRF and SSVM		DDSM and INbreast	10.0% loss

**Table 4:** Summary of reviewed works on deep learning models.

Subcategory	Related works	Year	Technique	Filter	Database	Evaluation metric
DL	[160]	2015	CRF		INbreast and DDSM-BCRP	89.0% of Dice
DL	[157]	2018	Adversarial FCN-CRF		INbreast and DDSM-BCRP	97.0% accuracy
DL	[158]	2018	FrCN		INbreast	92.97 segmentation accuracy, 92.69% Dice and MCC of 85.93%
DL	[174]	2018	CRU-Net		INbreast and DDSM	93.66% of Dice for INbreast and 93.32% for DDSM
DL	[161]	2019	ResCU-Net and MS-ResCU-Net		INbreast	91.78% of Dice, 94.16% accuracy, and Jac of 85.12% based on MS-ResCU-Net
DL	[162]	2019	U-Net and AGS		DDSM	82.24% *F*-score, 77.89% sensitivity, and 78.38% accuracy
DL	[169]	2019	RU-Net	cLare filter	INbreast and DDSM-BCRP	98.0% of Dice, 94.0% of IOU, and 98.0% accuracy
DL	[170]	2019	U-Net	Laplace filter	DDSM	97.80% of Dice and 98.50% of *F*_*i*_-score
DL	[171]	2019	AUNet		INbreast and DDSM	81.80% of Dice for DDSM and DI of 79.10% for INbreast
DL	[163]	2020	Mask RCNN		INbreast	88.0% of Dice
DL	[164]	2020	FrCN		INbreast	92.69% of Dice, 92.97% accuracy, and Jac of 86.37%
DL	[165]	2020	U-Net	Adaptive median	INbreast and DDSM	89.0% of Dice and mean IOU of 90.90%
DL	[159]	2020	DS-U-Net	cLare filter	INbreast and DDSM	82.7% of Dice, Jac of 99.7%, and accuracy of 83.0%
DL	[166]	2020	cGAN	Median filter	INbreast	88.0% of Dice, Jac of 78.0%, and 98.0% accuracy
DL	[167]	2020	cGAN	Morphological filter	DDSM	94.0% of Dice and IOU of 87.0%
DL	[168]	2020	Mask RCNN and DeepLab	Savitzky Golay filter	MIAS and DDSM	80.0% accuracy
DL	[175]	2020	Mask RCCN-FPN		DDSM	91.0% accuracy and 84.0% precision
DL	[176]	2020	U-Net		DDSM	79.39% of Dice, AUC of 86.40%, and 85.95% of accuracy
DL	[172]	2021	U-Net		DDSM	88.0% accuracy
DL	[173]	2021	U-Net		MIAS and DDSM	98.87% of Dice, AUC of 98.88%, and *F*_*i*_-score of 97.99%

## Data Availability

It is a review paper; hence, the data used is from previous published works.
